# “Self-testimony of a Past Present”: Reuses of Historical Film Documents

**DOI:** 10.1007/s00048-021-00300-z

**Published:** 2021-05-12

**Authors:** Anja Sattelmacher

**Affiliations:** Institut für Musik- und Medienwissenschaft, Humboldt-Universität zu Berlin, Georgenstr. 47, 10117 Berlin, Germany

**Keywords:** Visual history, Scientific film, Research film, Film provenance, Re-use, Political education, Visual History, Wissenschaftlicher Film, Forschungsfilm, Filmprovenienz, Reuse, Politische Bildung

## Abstract

Has the history of film digitization ever been incorporated in questions of evidence and knowledge production? The digitization of thousands of films from the former Institute for Scientific Film (IWF) that is currently underway gives an occasion to think about the provenance and reuses of filmic images as well as the ways in which they claim to produce scientific (or in this case, historical) evidence. In the years between 1956 and 1960, the German Social Democrat, historian and filmmaker Friedrich “Fritz” Terveen initiated a film series that used historical found film footage in order to educate university students about contemporary history. The first small series of films was entitled *Airship Aviation in Germany* which consisted of four short films using found footage of zeppelin flights, of which the earliest images stem from around 1904 and the latest from 1937, the moment of the “Hindenburg disaster.” This article explores how Terveen sought to shape the political landscape of history teaching in the new Federal Republic of Germany by first setting up nation-wide visual archives to host historical film documents, and secondly by seeking to improve the political education of a new generation of young Germans with the aid of the moving image.

## Abstract (de)

Wurde die Geschichte der Filmdigitalisierung jemals in Fragen der Beweis- und Wissensproduktion einbezogen? Die gerade stattfindende Digitalisierung von vielen Tausend Filmen des ehemaligen Instituts für den Wissenschaftlichen Film (IWF) gibt Anlass, über die Provenienz und Wiederverwendung von Filmbildern nachzudenken als auch über die Art und Weise, in der sie behaupten, wissenschaftliche (oder in diesem Fall historische) Evidenz zu produzieren. In den Jahren von 1956 bis etwa 1960 initiierte der deutsche Sozialdemokrat, Historiker und Filmemacher Friedrich „Fritz“ Terveen Filmreihen, die mit vorgefundenem Filmmaterial Hochschulstudent*innen über Zeitgeschichte aufklären sollten. Die erste kleine Filmreihe darunter war *Luftschifffahrt in Deutschland*, die vier kleine Filme mit gefundenem Filmmaterial über Zeppelinflüge umfasste, von denen die frühesten Bilder aus der Zeit um 1904 und die letzten aus dem Jahr 1937 stammen und die „Hindenburg-Katastrophe“ zeigen. In diesem Artikel soll gezeigt werden, wie Terveen die politische Landschaft des Geschichtsunterrichts in der neu gegründeten Bundesrepublik Deutschland zu gestalten suchte, indem er erstens danach strebte, ein bundesweites Bildarchiv mit historischen Filmdokumenten einzurichten und zweitens die politische Bildung einer neuen Generation junger Deutscher mithilfe des bewegten Bildes verbessern wollte.

## Introduction^1^

“Why do I have to bother watching this, even today”? This is the title of a reader’s commentary on a book review of the recent publication *Visual History of National Socialism* by Gerhard Paul in the German daily newspaper *Frankfurter Allgemeine Zeitung* (FAZ), implying that perpetuating and re-editing visual material of former Nazi propaganda only leads to new problems with regard to the (re-)circulation and (re-)idolation of these images (Zimmermann 2020). This reader’s comment reflects a notorious worry that accompanies the presence of visual material from the era of the National Socialist regime between 1933–1945 in television, newspaper, video-channels or chat rooms. This concern becomes even more uncomfortable today, as an ever-growing number of film clips and photographs are digitized and made available through online platforms. Looking at digitization processes and the discussions linked to them, it seems that similar concerns about misuses surfaced in the years before and after 1960, when films from the Nazi era were aired on television, coinciding with a growing consciousness for the recent German past (Keilbach 2010). This article looks at how film footage of historical events from the years 1904–1938 was reused in educational contexts and provoked questions about how to treat the past through film. Previous studies have already discussed the epistemic role of scientific films and have highlighted the ways in which visual representations can be problematic for the creation of scientific evidence (i.e., Cartwright; Cartwright & Sturken 2009). They have also explored how, within the field of history, film must be seen as a problematic source (Riederer 2006). In recent years, scientific film has repeatedly become the subject of studies within the field of the history of knowledge. Particularly noteworthy are the works of Lisa Cartwright, Scott Curtis, Oliver Gaycken, Hannah Landecker and others (for example, Cartwright 2011; Curtis 2015; Landecker 2011; Wellmann 2011; Gaycken 2015). Others have focused on the role of educational films (Orgeron et al. 2012; Bonah et al. 2018). In most cases, the focus has been on the creation of pre-war scientific films from the natural sciences.

Not many studies have looked at historical film footage that was re-edited for scientific purposes and that was intended to produce knowledge within the discipline of history and the history of science and technology (e.g., Boon 2008; Acland & Wasson 2011). Hence, this article constitutes an attempt to approach historical films which reflect the time of the “Third Reich” from the perspective of the early (West German) post-war period. Central to this text is the question of how early undertakings in visual history were implemented within school and university curricula and how film footage could offer a way to critically look at the recent past. This study is occupied with the period shortly after the Second World War, focusing primarily on the years between 1952 and 1960. It marks a time in which the confrontation with the recent past through the medium of film was highly controversial for most Germans (Hahn 1997; Fisher 2010; Verheyen 2010). The corpus of films looked at here stem from a series called *Film Documents of Contemporary History* (*Filmdokumente zur Zeitgeschichte*), which is housed at the German National Library of Science and Technology (TIB) in Hanover and is partially accessible online.^2^

## The Institute for Scientific Film in Göttingen

Between 1957 and 1959, the historian Friedrich Terveen published a film series entitled *Airship Aviation in Germany* (*Die Luftschiffahrt in Deutschland*) at the Institute for Scientific Film (*Institut für den Wissenschaftlichen Film*, hereafter IWF) in Göttingen.^3^ At that time, the IWF had just recently been established. It was founded in 1956 by the German engineer Gotthard Wolf as a successor institution of the former Reich Institute for Film and Images in Science and the Classroom (*Reichsanstalt für Film in Wissenschaft und Unterricht*, hereafter RWU).^4^ The foundation of this institute was marked by both discontinuities and continuities. The RWU had been crushed by the Allies and some of the film material had been confiscated as spoils of war (Tolle 1961: 88). After its reestablishment as the IWF, the institute concentrated exclusively on the production and distribution of research films for science. Until its closure in 2010, it produced around 11,500 films from various academic disciplines; the fields of biology, ethnology, technical sciences and medicine were all represented. The founder and director of the IWF, Gotthard Wolf, claimed to be aware of the manipulative and illusionary effect the films had had in the past and now wanted to reestablish the institute on the foundation of exact scientific methods.^5^ In contrast to its sister institution, the Institute for Film and Image in Science and Education (*Institut für Film und Bild in Wissenschaft und Unterricht*, FWU) which distributed film for use in primary and secondary schools, IWF films were made available exclusively for research and teaching at universities (Terveen 1958b).^6^

Despite the apparent ruptures with the Nazi era, continuities among personnel and institutional decision-making persisted. For example, Gotthard Wolf, who had been the director of the Technical Research Film Department at RWU, became the first director of the IWF (Kühn 1998: 53). Then there was the figure of Konrad Lorenz, already a well-known ethnologist and member of the NSDAP in the 1930s, who made a decisive contribution to the founding of the IWF in the 1950s (Wolf 1967: 46; Scholz 2019: 3). There is also great overlap between the film stock of the IWF and the RWU. Large parts of the medical, military, agricultural and folklore recordings were taken over, in addition to a collection of personality recordings and contemporary historical records, such as speeches and public appearances by public figures since 1895. This stock also included the source material from which Terveen compiled his series on zeppelin flights. These were four very short film clips which showed different types of airships on their various flights, beginning with the first flight of the LZ 3 and ending with the Lakehurst disaster of the LZ 129 in 1937. The material was taken from contemporary footage—some of it had been produced by private film companies which was then purchased by the IWF, some of it was from weekly newsreel footage (*Wochenschau*) and was thus obtained after the Second World War by the Federal Film Archive. In particular, the film footage of the explosion and crash of the LZ 129 Hindenburg on May 6, 1937 in Lakehurst has entered the cultural memory of aviation history through its distribution via newsreels and later via documentation and excerpts in museums (Frank 2008). The series *Airship Aviation in Germany* is the subject of this study; in particular it focuses on two of its films—the first and the last ones (named by their signatures: “G 24” and “G 41”). Based on this film material, the following questions are to be discussed: how did historians of the war generation view historical film recordings of the recent past and how were questions of usability and reception of the films negotiated? What sort of understanding of historical evidence did historians like Fritz Terveen have, and what were their expectations of the films? It becomes apparent that there were divergent views within the IWF, which led to a renegotiation of the historical-critical content of the films by changing accompanying texts and metadata of the films.

## Films as Encyclopedic Units

The series *Airship Aviation in Germany* was part of a larger film collection at the IWF, entitled *Film Documents on Contemporary History* (*Filmdokumente zur Zeitgeschichte*). This title is indebted to the institute’s concept of producing “documentation films” (*Dokumentationsfilme*). This term was deliberately chosen by Wolf, as opposed to the usual term “documentary films,” implying that these films documented processes of nature, history, social life or technical approaches in a style similar to the way in which a scientific observer in the field or in the laboratory does (Wolf 1967: 11). As Gotthard Wolf stated on many occasions, the intention was to fix and preserve a process with the camera and make it visible at the same time. In his understanding, a documentation film was a product of a complex process that consisted of measuring light, optics and velocity, thereby collecting all the relevant cinematographic data about a movement (cf. Schulze & Waltenspül 2019). The raw data that came with each film, i.e., the uncut material of the film, was to be archived for later scientific purposes. In this way, Wolf attributed to the films made at his institute a “realness” (Wolf 1967: 171) that distinguished them from documentary films; the films stemming from the IWF, as he stated, were just “copies from the world as it is” (Wolf 1967: 172). Nevertheless, the IWF films were full of allusions and left room for different interpretations. As Tania Munz has meticulously shown, especially the films by Konrad Lorenz, who was deeply involved in the foundation of the IWF and contributed many films to its collection, show various examples of manipulations, tricks and suggestions that were necessary to make the films more credible and also more legible (Munz 2005: 58–65). Meanwhile, the idea of using film as a historic or scientific document is as old as the history of the medium itself (Wöhrer 2015). Within the studies of early cinema, it is commonly observed that non-fictional films that were made before World War I are rather descriptive in character, whereas the documentaries produced from roughly 1918 onwards have a rather hermeneutic function (Gunning 2015: 158–159). Terveen might have had this distinction in mind when he emphasized that his films had nothing to do with interpretative documentaries (for instance Terveen 1960a). *Airship Aviation in Germany* consisted of many different clips from news reels shot at different places that he tried to put into a didactic compilation to create a retrospective view of the history of airship aviation in Germany.

Friedrich “Fritz” Terveen had been a lecturer in history (initially art history) at the IWF since its founding (Forster 2015: 351–352). He began his work there in 1953 and left the institute in early 1960, shortly after completing the series on airship travel in Germany. Like Wolf, Terveen ascribed a particular value to the film as a document: he noted that there was a lack of understanding of how to use the film as a scientific source for research and university teaching. In his view, the task of historical research films was to document past events and to make them available in such a way that a variety of scientific questions could be discussed based on the material. For this reason, Terveen thought it would be helpful to categorically exclude feature films and narrative documentaries from his collection (Terveen 1960a; Ebbrecht-Hartmann 2015). Without explicitly mentioning it, it seems that Terveen (and Wolf) referred to a distinction first made by the British filmmaker John Grierson in the 1930s by claiming that the documentary film was qualitatively different from formats like news reels, scientific films or educational films (Grierson 1976 [1932–34]). For Grierson, documentary-making was set apart from the other forms mentioned above because it was able to be selective, interpretative and creative (Grierson 1976 [1932–34]: 21). Terveen’s aim, however, was to create a cinematographic tradition using film material in the same role that the document, the seal, the clay tablet, the palimpsest or other types of sources play in other contexts: that of unmediated material without any form of interpretation. With the help of film as a medium that could be reproduced and screened at any time and in any location, he sought to create a new source genre for the future historian to enable the study of movements, gestures and postures, thus enabling historical-biographical studies without necessitating visits to archives or interviews (Terveen 1960b: 279). The kinds of films envisioned by Terveen (and also Gotthard Wolf) were considered by Grierson to be “lecture films” that do not dramatize, but only describe, and, “in any aesthetic sense, only rarely reveal” (Grierson 1976 [1932–34]: 29). For Grierson, this quality represented a major limitation of the format, whereas for Terveen in particular, and for the IWF in general, the lack of dramatization was an important feature.

In the institute’s early years between 1953 and 1958, the film series *Film Documents on Contemporary History* (*Filmdokumente zur Zeitgeschichte*) was part of the IWF’s large-scale project *Encyclopaedia Cinematographica* (EC). The basic idea of this encyclopedia of films was to capture in film all forms of life, movement and behavior in nature and even in technical processes.^7^ A scheme was created which every film had to follow (Wolf 1967: 15–170). To fit into this scheme, films had to have a certain form. They had to be divided into small subunits that could be analyzed, possibly recombined and compared with each other. The EC covered the fields of biology (divided into the subdisciplines of microbiology, botany and behavioral science), ethnology and folklore, and technical sciences. Initially, history was also included as a separate subject area before it was converted into the independent series *Filmdokumente *in 1959. As for the historical film documents, initially, only “valuable” original recordings of events and personalities from the years 1895–1933 were published for use in research and university teaching (Terveen 1956: 3). Among the first films in this series were re-edited recordings of the former *President of the Reich*, Paul von Hindenburg, as well as of the physician Ferdinand Sauerbruch in his operating room at the Charité hospital. The films on airship travel in Germany can also be found among these early recordings, with two of the four films in the series containing material from the National Socialist era beginning in 1933.

**Fig. 1:**
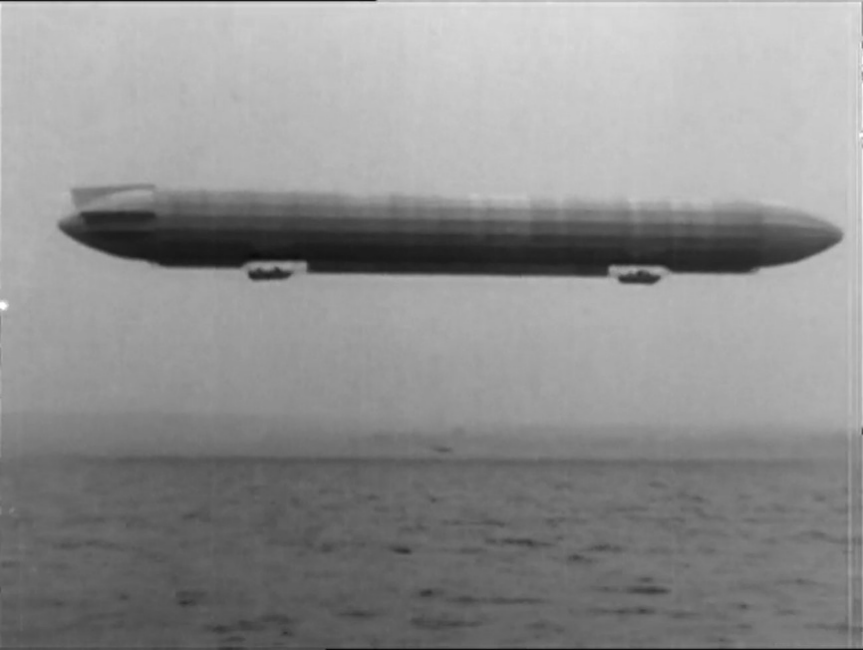
Scenes of the Airship LZ 3 (Z1) leaving its hangar in Manzell/Germany and flying over Lake Constance. Fritz Terveen, Zeppelin-Luftschiffe 1906–1910 (G 24), IWF 1957. Provided by the TIB AV-Portal, b/w, 6’39. 10.3203/IWF/G-24. Courtesy of the TIB Hannover

If we take a closer look at the earliest film in this film series, *Zeppelin-Luftschiffe *1906–1910, signature G 24, we see the sequence of numerous mounted image sequences.^8^ The film proceeds through various stages of zeppelin history, moving forward chronologically according to the construction number of the respective zeppelin and subdivided into different target flights. Each sequence shows a different zeppelin flight. Thus, the film begins with the flight of the model LZ3, which seems to float out of the construction hall directly over the lake (see Fig. 1).

**Fig. 2:**
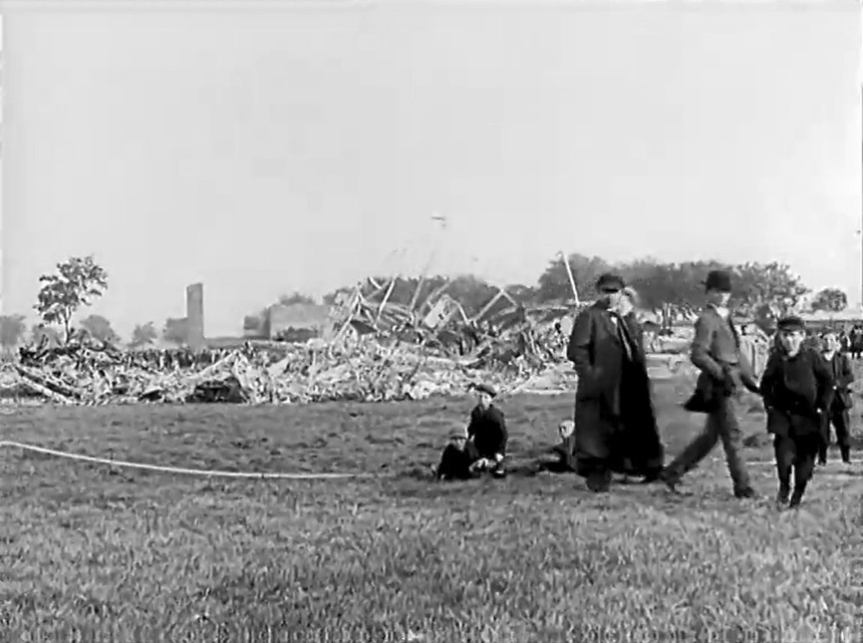
A scene showing the wreck of the crashed air ship LZ 4 in Echterdingen on August 5, 1908. Terveen, Zeppelin-Luftschiffe 1906–1910 (G 24), IWF 1957. Provided by the TIB AV-Portal, b/w, 6’39. 10.3203/IWF/G-24. Courtesy of the TIB Hannover

This is probably the earliest recording of a zeppelin. The following shot shows the next model, the LZ 4, landing in Echterdingen on August 5, 1908. In this clip, a cheering crowd greets the airship. The film alternates between close and distant shots of people and the flying object. Next cut, next event: the viewer is transported to the site of the catastrophe of Echterdingen on the same day. Children look at the wreckage of the airship, which had been cheered by the crowd only moments before, people walk by, the camera moves from right to left in the picture (see Fig. 2).

**Fig. 3:**
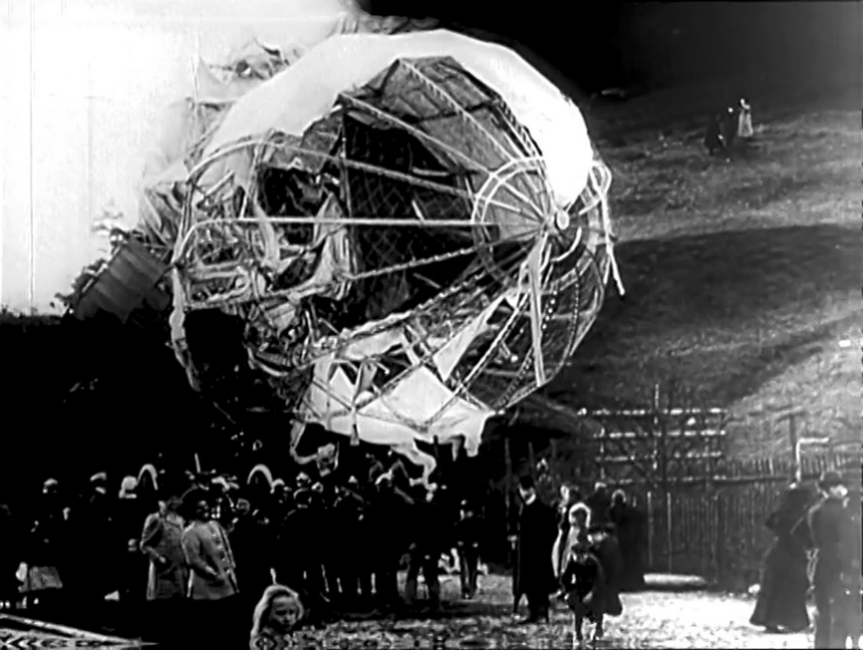
Still from the moment after the airship crashed, showing the wreck of LZ 5 in Weilburg on May 10, 1910. This still is enigmatic in the way that it seems to merge different images from different scenes or even films together. Terveen, Zeppelin-Luftschiffe 1906–1910 (G 24), IWF 1957. Provided by the TIB AV-Portal, b/w, 6’39. 10.3203/IWF/G-24. Courtesy of the TIB Hannover

The next sequence shows the accident of the LZ 5 near Göppingen on May 30, 1909; this is immediately followed by a shot of the landing of the LZ 5 in Frankfurt in which the flying airship is again received by crowds of people. Another sequence shows the landing of the zeppelin LZ 6 on a firing range in the presence of Kaiser Wilhelm II on August 29, 1909. Again, shots of the crowd and of the zeppelin alternate. The last sequence shows the LZ 5 after the catastrophe of Weilburg on May 10, 1910 (Fig. 3). Once again, the remaining skeleton of the zeppelin is shown as men bring down the surrounding tarpaulin.

As we have seen, this film about zeppelin flights has an extremely rigid structure, contains almost no narrative elements, no temporal flashbacks, is rather monotonous in its camera angles and features quite a lot of cuts. This is partly due to the nature of the material, as it stems from documentary film footage from the early years of film, which was then reassembled in 1957, at which point Terveen published it. Furthermore, this film composition also had to fit into the logic of all films made for the EC project. These EC films—which showed scenes of the fighting behavior of male fish, life in indigenous cultures, as well as the growth of plants or the movements of steel when cut into pieces—were all structured according to a modular system, using a narrow framework for camera guidance, editing and lighting. Animals, if they were present in the film, were never trained and a dramaturgy was generally avoided. The film units of the EC were intended to function as small data packages: they needed to be comparable with each other, searchable, exchangeable and at the same time part of a larger system (Reichert 2009). The “Instruction Sheet on the Use of Historical Film Documents” (*Merkblatt zur Verwendung der historischen Filmdokumente*) on the use of historical film documents from the *Encyclopaedia Cinematographica* from 1956 gives an idea of what it meant to fit historical found footage material into the rigid structure of the EC: “Each of the film units presented in the EC […] has been worked out as an individual source piece, for which a reference to the other historical sources and statements must be made or taken into account in order to clarify the facts contained therein.”^9^ The expectation that historical footage structurally underwent a certain biologization also becomes apparent in the rhetoric used by Terveen. An early text speaks of the fact that the film itself could become a “preserved specimen” of the scientist, which would serve for further research (Terveen 1960a: 3). These were almost exactly the same words that Gottfried Wolf had used at the commencement of the EC. Wolf understood scientific film as a “permanent preparation of movement” (*Bewegungsdauerpräparat*), which recorded movement in a broader sense and enabled analysis (Wolf 1967: 16). In this sense, the film strip itself was supposed to serve as a specimen that could be looked at repeatedly at any time, just like the organism under the microscope in a laboratory. Narration, condensation or simplification through film was to be avoided at all costs. Rather, by producing many individual, thematically closely-related historical film units and then creating combinations and variations based on them, producers strove to “draw on them to investigate differentiated questions.”^10^ It is remarkable that these developments took place without any obvious collaboration with filmmakers in France and the United States who—thanks to 16 mm film and lightweight camera technology—found ways of capturing scenes outside of the studio and in the field. This new form of documentary, called *direct cinema* or *cinéma verité*, claimed to develop a critical position towards the filming of so-called reality and required practitioners to actively become involved with the subjects with whom they dealt. In an echo of Gotthard Wolf’s statements, practitioners of direct cinema pursued truths with the help of film (Beyerle & Brinckmann 1991; Saunders 2007). Historians can only speculate as to why Wolf never mentioned other types of documentary making such as direct cinema. It is possible that he wanted to claim the EC as something unique and therefore omitted similar ideas in this context.

The EC created a system of references in which intertitles and accompanying booklets were linked both between films and between individual film sections. Terveen repeatedly emphasized the significance of the film as a source-like document. Just like the film units of the EC, they were conceived as small data packages which were to be made contingent and interchangeable, searchable and capable of being analyzed. Once more, this close linkage to the biological sciences becomes obvious in the leaflet cited above:

For the study of history, the significance of the cinematographic film recordings published in the *Encyclopaedia Cinematographica* lies in the fact that with their help, real film recordings of historically significant persons, events or locations from the period from 1895 to the present day and further into the future can be recorded and presented for scientific consideration.^11^

The leaflet further states: “The source material found—in any case, it is real footage, not feature film footage staged or staged in the studio—was not originally produced for scientific purposes. As a rule, it has so far rather been film footage with a publicistic character.”^12^

However, Terveen soon discovered that found footage of historical events could not be treated in the same manner as biological or technical film sequences showing different kinds of movements. His conclusions diverged from those of Gotthard Wolf with regard to the effect of the EC. Terveen doubted that the project could actually be useful for research purposes: “The Encyclopaedia of today is a collection of mostly technically inadequate, often unsatisfactory motion picture sequences, which in their present form will in the long run satisfy neither research nor teaching.”^13^ Ultimately, the history films were removed from the EC in 1958 and became a series of their own: *Film Documents of Contemporary History*. This series contains over 250 films on a wide range of topics. During his tenure at IWF, Terveen started a series within the *Film Documents *that he called *Personal Recordings* (*Persönlichkeitsaufzeichnungen*) which consisted of interviews with renowned artists, scientists and public figures (Forster 2015). The early interviews followed the rigid structure of EC films. They were rather small film units, showing the interviewee in only one position, speaking in a monologue directed at the camera. This format was continued by his successor at the IWF, Karl-Friedrich Reimers, and changed only slightly over time.

## Text-Image Relations in Historical Film Documents

A central component of every film published by the IWF was the accompanying leaflet, which contained important information about each film. For the film *Zeppelin Luftschiffe, 1906–1910* (iwf signature g 24), the accompanying script, which Terveen had created himself, was also of great importance for the audience’s perception of the film. It contains detailed background information on the origin of the material, the editing of the sequences and descriptions of the image content (Terveen 1958a). Since the film itself does not contain any explanatory comments, but instead relies on intertitles to indicate the model types of the airships and place names, the accompanying text plays a central role when it comes to the historical classification of the material, the contextualization of the images in relation to each other, and the origin of the photographs. When Terveen spoke of the “truth content” (Terveen 1956: 3) of a scientific film document, it was clear that the accompanying publication was implied as well. What Terveen meant by “truth” was actually the idea of historical evidence—in this case, the explanation of contemporary historical references—that film footage material could reveal. Looking at the published brochure that came with the film *G 24*, it becomes obvious that Terveen had meticulously researched the provenance of the material. In the publication we learn that the entire image material shown in the film was purchased by the IWF from the Albert Fidelius film collection in 1955 and then reprocessed by Terveen.^14^ Fidelius was a film collector and businessman from Berlin, whose stock comprised an estimated 700,000 normal film positives, including silent Eiko, Messter and sound newsreels until around 1950. In 1962, the Berlin Senate bought up the entire stock and left it to the Deutsche Kinemathek (König 2015).

**Fig. 4:**
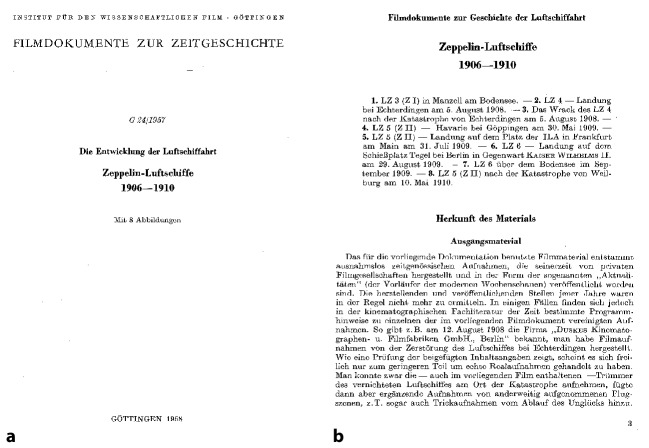
**a**,**b** Title page and first page from the accompanying publication for the film *Zeppelin* *–* *Luftschiffe* 1906–1910 (Terveen 1958a)

The shots for the zeppelin films, which Terveen took over from Fidelius, first had to be copied from 35 mm normal film to 16 mm film, as the latter was the usual film format that was distributed to schools and universities. As Terveen himself wrote, he was unable to extract much historical information from the source material itself, which is why he had to carry out painstakingly detailed research into the information on flight locations and object types and then label it in the newly edited version (Terveen 1957: 4). In addition, the source material was heavily damaged due to in part to improper storage. As a result, the film had shrunken and had to be regenerated by a special process in order for it to be able to be copied at all. The image effect was severely impaired by the numerous cuts, aging, and copying of the material. Not least because of the poor image quality of the film material, the accompanying text played a decisive role in the reception of the film. Following the source evaluation, Terveen gives a detailed description of the images in his text, which he partly supplements with the origin of the footage as well as extensive technical details about the airship shown in each image (Terveen 1958a: 4). Following the image descriptions, Terveen provides a brief historical outline of the history of the zeppelin airship in his text, as well as reprints of selected sources from the Zeppelin Archive (Fig. 4). What is completely missing in Terveen’s texts about the films, as well as in all later published texts, is a critical examination the images themselves. The texts do not discuss what effect the films might have on a contemporary audience (notably history students) or how they might have affected people who first saw these films in the past. The images’ historical contexts and a discussion of the contemporary political era are also omitted. Thus, aside from learning about their archival provenance, the reader does not gain insights into the historical background of the images. Instead, the audience encounters only detailed information about each airship model. So, while we may see a series of short sequences that recall scenes of the *cinema of attractions* from the early nineteenth century as described by Tom Gunning (Gunning 1990), the text ignores this cinematic relationship in favor of delivering deep insights into the provenance of the footage, as well as on technical details about the zeppelins and their construction. The combination of text and image, it seems, forms a unit which delivers knowledge on technical aspects of the history of airships as well as on the history of early film archiving. The film itself seems to have only an illustrative function and serves to accompany the text, and not, as intended by Terveen, vice versa.

**Fig. 5:**
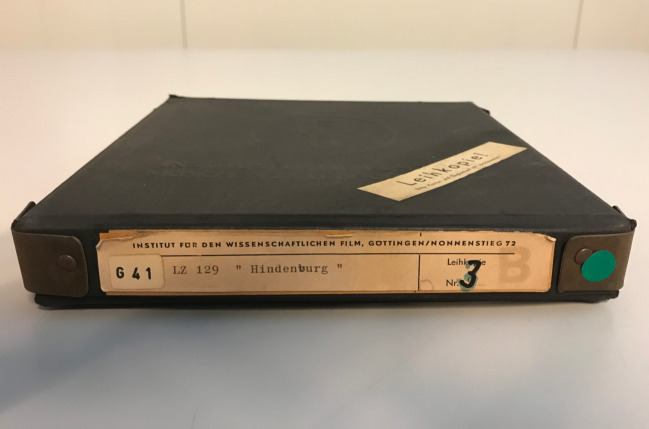
A film box of the film G 41: LZ 129 “Hindenburg” by Terveen from 1958. The number “3” written on the box indicates how many times a new copy was made to replace the film as result of deterioration and wear. © Miriam Reiche, TIB Hannover

The film and text together form a source-critical edition that was intended exclusively for distribution at universities. Each film box, like the one shown in Fig. 5, carries a number, which reveals how often a film went back and forth between the IWF and the copying laboratory. The more a film was used (by borrowers), the more copies of it the IWF had to order. The number on the film box of the film with the signature *G 41 – lz 129 “Hindenburg”* indicates that the film was not among the ones often borrowed from the IWF.

However, according to the statistics, the *film documents* were among the most sought-after films—at least until 1960, the year when Terveen left the institute. Even though there were no overall statistics kept on the distribution and sales of historical films alone, there was a tendency for demand for these to increase over time. For example, a statistical report from 1959 shows that out of a total stock of 26 film documents, a total of 88 uses were made of them between 1955 and 1959, which corresponded to 70% of the use of the total stock.^15^ Only half of these users, however, came from a purely academic environment, the other half consisted of users from radio, television, the Educational Film Hire Services* (Landesbildstellen)*, the FWU, and other universities and educational institutions. For Terveen, this was a clear signal to make the series accessible to groups outside the universities. The figures also show that the IWF slowly abandoned its goal of distributing films for exclusively research purposes.

But where did the *film documents* get their visual evidence, or the sense of “truthfulness” to which Terveen referred? The early films about airship travel shared neither an aesthetic nor a narrative approach. The film *Zeppelin Luftschiffe 1906–1910* (g 24) consists of a montage of individual film snippets, laboriously held together by short intertitles. As Gregg Mitman and Kelley Wilder have argued, the power of (film) images lies in their indexicality, or the fact that they are integrated into a system of references, for instance by being archived and thus retrievable, referenceable and citable (Mitman & Wilder 2016: 12–15). This was also the goal of Terveen’s work. Since the early 1950s, he wrote texts on the systematic creation of a scientific film archive using historical film material. His idea was that all archives throughout Germany should collect and index their documentary film holdings and make them available at a central “collection point” (Terveen 1955b: 173). In contrast to the historical and political film material already collected by the Federal Archives, Terveen was thinking primarily of the many regional archives with their regional historical material. However, due to the fact that film wears out very quickly with use and that it was often difficult to filter out the desired individual document from a large amount of film meters, this project proved a difficult undertaking. It therefore made sense for him “to remove special source versions from the archived material and to pass them on to researchers in the university, seminar and research institute, divided into individual complexes, precisely dated and carefully analyzed in terms of content.” (Terveen 1955b: 174) Only Terveen’s private correspondence reveals that he had imagined the IWF as just such a collection point. Terveen had made suggestions to transfer the IWF to such a collection point, but those remained unheard by Wolf.^16^

## The Impact of Filmic Images

The timing of the release of the series *Airship Aviation in Germany* in 1957/1958 marks a turning point both for Terveen personally and for the policy of dealing with the recent German past at the IWF. While the two previous zeppelin films contained material from the years after 1933, Terveen began to process and edit film material available in the Federal Archives that showed the political actors of the so-called “Third Reich.” He edited speeches by Adolf Hitler, Hermann Göring, Wilhelm Frick, Ernst Röhm, Joseph Goebbels and others and reissued the newsreel on Hitler’s 50th birthday together with an accompanying publication (Terveen 1960c). At the same time, the series *Film Documents* was separated from the EC, based on Wolf’s argument that he did not want to show this material at international meetings where the EC films were often presented.^17^ Wolf had issued a kind of decree that all material containing footage from the Nazi era should be shown exclusively for research purposes at the university, but under no circumstances should it be screened in the United States. Terveen’s attempt to show the material in educational institutions outside the university was openly rejected by the IWF advisory board, which included Kurt Zierold, one of the leading officials of the RWU.^18^ As the statistics shown above and the newspaper articles quoted in the following show, there was great public interest in these films. In 1960, the director of the *Landesbildstelle* in Hamburg, Fritz Kempe, published an article in the magazine *The Youth* (*Die Jugend*) entitled “Should We Show Hitler to the Youth?” (Kempe 1960: 6ff.) The author, who was an acquaintance of Terveen’s, came to the conclusion that this was an educational responsibility. He argued that it was absolutely necessary to show former propaganda films, newsreel footage and other film material from the Nazi era to young people in order to sensitize them to their past and to the ways and means by which the film can capture an audience’s attention and sympathies. Kempe was quite certain that the main reason for the previous reticence to showing Nazi material was that the older generation did not want to be confronted with the past.

One voice from the *Hamburger Echo, *a newspaper with a social democratic orientation, went even further and asked whether the films in the series *Film Documents on Contemporary History* were only intended for secret societies, or whether there was some other reason that the public had such difficulty seeing them (O.N. 1959). In view of a broad public interest and significant public financing, the author of the article found it incomprehensible that film documents on the history of the years from 1933 to 1945 would only be lent out against the submission of a written declaration that ensured that they would be used strictly for scientific or educational purposes. Finally, it should be remembered that in addition to the training of future history teachers, the films could be useful in education at adult education centers, film clubs and also at secondary schools. Terveen’s thinking had been moving in this direction for some time. He had come around to making historical documentary film accessible to a wider public, as he indicated in an essay from 1955:

To us today, the appearance of National Socialism is still familiar from our own experience. But what will this knowledge be like in 30 or 50 years? If it were possible to record for posterity the countless film strips of the Reich Party Congresses, the rallies, speeches, marches, assemblies, carefully dated and scientifically processed in every way, the future study of National Socialism, in particular its means of mass leadership and the hollowness of its speeches, would be a commendable service and would open up an additional source of understanding for the historian of that time. (Terveen 1955a: 65–66)

Of course, these sources needed to be screened and explored by experts beforehand. The IWF had a very important role to play in this respect: in 1946 it was commissioned by the military government in Hamburg—still operating under the Research Department of the Institute for Film and Image—to clean up the film archives of all image locations in the British zone in accordance with the censorship lists that were issued by the British Allies.

The copies of all films that have been definitively or provisionally banned were collected and viewed at the Institute. […] The politically objectionable scenes in the provisionally banned films were removed. In accordance with the approved new versions, 3,700 copies were re-cut, given new titles and seals, and returned to the picture departments for distribution. (Schmid 1958 [1948]: 216)

Film material from the Nazi era had thus already been subjected to censorship by the Allies; by no means could all of the scientific films that had been produced between 1933 and 1945 be shown. With regard to the reception history of the films from the series *Film Documents*, in the late 1950s Terveen sent out questionnaires to student working groups asking them to provide information on whether the film they had ordered was suitable as a working tool and how the films were to be assessed in connection with historical-political instruction. In addition, he asked whether the film used was examined in isolation or rather compared with other sources. Finally he also solicited criticism and suggestions for improvement. The responses to these questionnaires revealed that, generally speaking, students welcomed the films as a further aid in historical-political lessons. However, Terveen also received much criticism with respect to the presentation of the films themselves. Respondents often complained that the material seemed too incoherent and fragmentary to form a comprehensive impression of the events. Terveen replied that the film documents were often not self-contained films, but rather “remnants of tradition” that were not further processed because this would be an intervention into historical research (Terveen 1960d: 16–17). One of the frequently mentioned concerns of the recipients of the film documents was the “revival of Nazism” through what was shown. Terveen saw no danger here. At least among the younger generation, he argued, the material was now viewed from a clear historical distance “in the midst of a changed political world.” (Terveen 1960d: 18)

## To Show or Hide Sensitive Material?

**Fig. 6:**
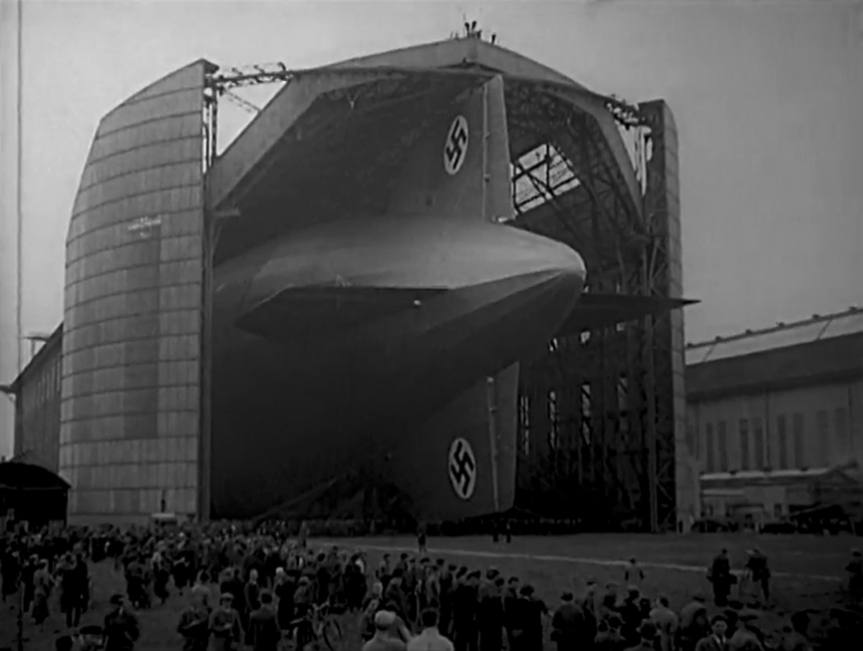
The airship LZ 129 coming out of its hangar in Friedrichshafen accompanied by thousands of people. These images were useful propaganda material for the National Socialist party under the “Third Reich.” Terveen, *LZ 129 “Hindenburg”* (G 41), IWF 1959. Provided by the TIB AV-Portal, b/w, 9’55. 10.3203/IWF/G-41. Courtesy of the TIB Hannover

Could Terveen use historical film documents to create scientific evidence about what happened while limiting the appeal of its propaganda? (Cf. Riederer 2003) Another film from the zeppelin series, which was made shortly after the first, but which featured images from the National Socialist era raises some doubts. This approximately 10-minute film, entitled *G 41 – Die LZ 129 “Hindenburg,”* follows a similar pattern to Terveen’s *G 24* film discussed above. This film also runs chronologically through the various stages of the model aircraft. It begins with shots from the ship’s hangar. About 100 men pull the airship out of the hangar in Friedrichshafen before it is ready for take-off (Fig. 6).

**Fig. 7:**
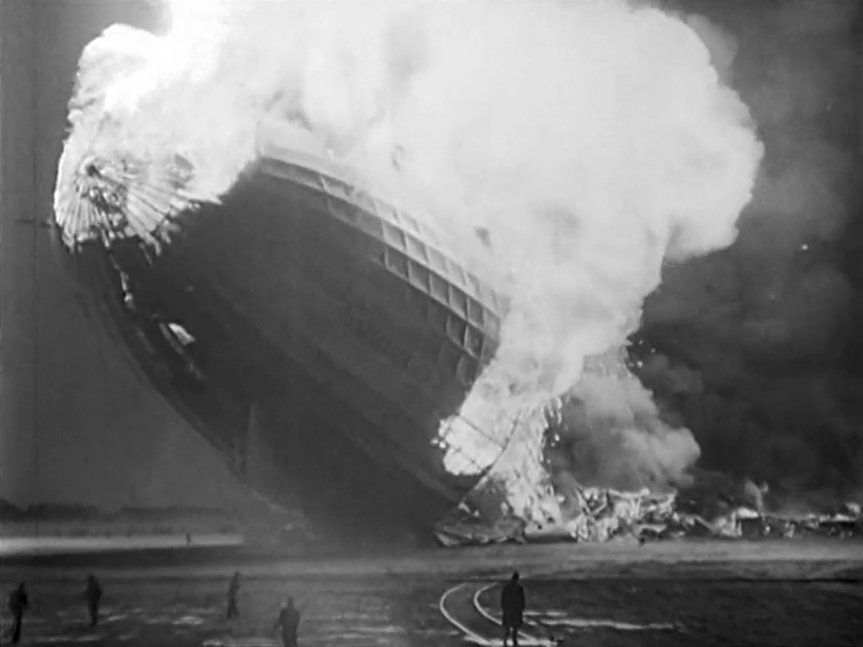
The airship LZ 129 burning and sinking on May 6, 1937. This event, known as the “catastrophe of Lakehurst,” is an iconic part of Europe’s cultural memory of the war and early days of airships. Fritz Terveen, *LZ 129 “Hindenburg”* (G 41), IWF 1959. Provided by the TIB AV-Portal, b/w, 9’55. 10.3203/IWF/G-41. Courtesy of the TIB Hannover

The swastikas on the ship’s wing are easily recognizable and repeatedly shown. This film is divided into the different journeys of LZ 129 *“Hindenburg.”* The take-off from Friedrichshafen is followed by footage of the flight to Germany from March 26–29, 1936, and the events and work of the engineers inside the airship can be seen. There are cheering people everywhere, and one sees cities such as Ulm and Berlin from a bird’s eye view. Then there is the flight over New York (no date) and finally the film ends with “the Lakehurst disaster” on May 6, 1937. The airship stands still in the air for a few seconds before a fire at the stern appears; finally, it sinks to the ground where it is consumed by flames (Fig. 7). There is also an accompanying text to this film, however it was written only in 1982, nearly 23 years after its creation, by the engineer Walter Rathjen, who had helped to build the air and space hall at the Deutsches Museum in Munich where he was the head of the aerospace department. In the accompanying text to *G 41* it says:

Although the Reich Minister of Aviation, Hermann Göring did not approve the airship as it has no use for military purposes and no value for the future as an efficient means of transport, the state had contributed a lot of money to the construction of the largest airship in the world, with the intention of advertising effectiveness of the world-famous zeppelins. A flyover of a zeppelin was a great event and has indeed has had an enormous effect. Children had a day off from school, factory sirens were wailing, the workday was interrupted, and people stood and looked fascinated and thrilled at this enormous, unique phenomenon in the sky. In this case, however, the majestic appearance was accompanied by a loudspeaker voice which called for the election of the National Socialist Workers’ Party. But also for the airship industry this trip was a valuable and necessary advertising. (Rathjen 1982: 4)

However, in the film, there is nothing to be heard of this loudspeaker voice mentioned in the text by Rathjen. The reason why he edited the film without sound is not entirely clear. Even stranger is the fact that the loudspeaker is mentioned in the accompanying text. This creates a gap between what is shown—without sound—and what is written, which refers to the sound recordings. The text completely refrains from a source criticism of the pictures; not even a reference to the original material—in this case the German newsreel (*Wochenschau*)—is given. It only appears in the film credits. The accompanying text by Rathjen, in turn, refers exclusively to the technical history of airship travel—explaining details of individual aircraft models and the journeys and the origins of the crash. Thus, the accompanying text does not supplement the film, nor does not classify it, but rather leaves the pictures and their impact to stand on their own. Rathjens’ text positions the film into the narrative of “great masterpieces” that was customary at the Deutsches Museum at the time, leaving the technical developments without social and political-historical contextualization (Königsberger 2009). It depoliticizes the described images and renders them interchangeable. This later framing did not correspond at all to Terveen’s intention. In an unpublished paper on the handling of film material from 1933 to 1945, he argues that special care should be taken when writing the accompanying text to the film document:

Since film recordings from the National Socialist era are a very complex means of propaganda, produced with all the means of a political and psychological direction that is highly refined and extremely well planned, we consider it absolutely necessary to go beyond pure documentation and to attempt to interpret the handling of the cinematic means, insofar as they determine the respective document. This is all the more indispensable as an intimate knowledge of the cinematic methods used here by the original producers cannot normally be expected even from the scientific user of such film documents.^19^

Terveen was aware of the potential impact of images, and he wanted the historically sensitive material to be published with the highest possible degree of editorial attention. At the same time, he trusted that the viewer of his time would have a critical detachment from the fact that he or she was part of the next generation that did not “step out of a brown outside world into the cinema and in front of the concentrated brown screen.” The viewers of his time could experience film as a “self-testimony of a past present.” (Terveen 1960d: 18) Nevertheless, Terveen’s actions lagged behind his ambitions, at least during his time at the IWF. There, critical voices of the Nazi past were not welcome, which was one of the reasons Terveen was happy to leave the institute in 1960.^20^

## Conclusion

About 15 years after Terveen had left the IWF to assume his position at the *Landesbildstelle* in Berlin, the latter printed a short publication entitled *Contemporary History in Film* (*Zeitgeschichte im Film)*. Interestingly enough, Terveen was not part of the editorial board, although the topics tackled in the book were compatible with the ideas he had developed for how to present images from the recent past (Bunk et al. 1974). This publication presented many ideas for how to approach the Nazi past in educational films. It contained short articles about the uses of film as a historical document and as a political instrument. The booklet meticulously analyzes scenes from different film examples from the era of National Socialism and also provides extensive further readings and primary sources. It tries to sensitize the readers’ eyes to how and why images have propagandistic power and introduces a media critique of the film as a historical document in a very structured manner. In this publication, each film in question was put into larger socio-historical context by including many details about the provenance and use. Such a strategy is a marked departure from that of the IWF’s accompanying publications. Finally, the publication of the *Landesbildstelle* posed fundamental questions to students (of secondary schools). It would seem that television in the 1970s had found a more reflective solution to present problematic film material to students than the IWF had in the 1950s. Of course, the political atmosphere in West Germany had undergone significant changes between 1957 and 1974. A broad social and intellectual movement within the country had revealed Germany’s problematic treatment of the Nazi past and sensitized the public; audiences were prepared to treat these films responsibly by performing critical evaluations of images depicting terror, war and prosecution (for example Kraushaar 2000; Klimke & Scharloth 2007).

**Fig. 8:**
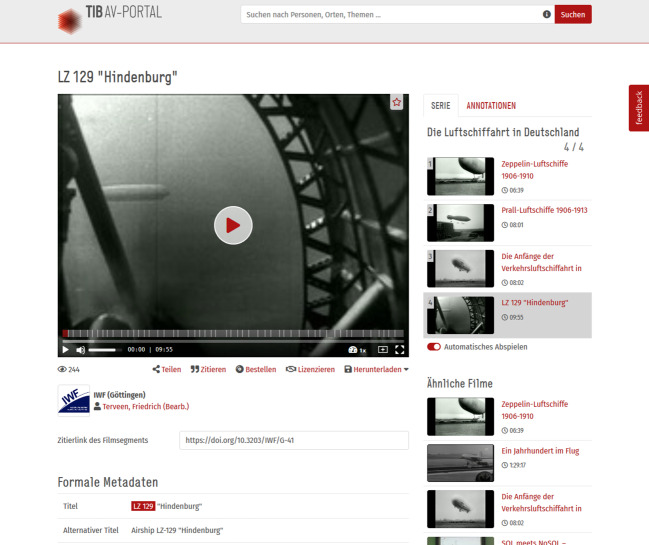
Screenshot from the TIB-AV-Portal, showing the film *LZ 129 “Hindenburg” *(G 41) by Terveen 1959, embedded in the context of the site with annotated information and metadata, see https://av.tib.eu/media/11212?hl=zeppelin. Screenshot reproduced with the permission of the TIB Hannover

Meanwhile in 2001, the IWF began its first digitization project, funded by the German government. The aim of the project was to improve the quality of “audiovisual knowledge” by increasing media competence as well as improving the supra-regional provision of the films (Beuers & Hanisch 2004). The series *Airship Aviation in Germany* was among the films that were chosen for digitization, along with the three other films from the series. As we look into the digitized film versions and the archival material that is connected to the genesis of the zeppelin films, it becomes clear that that the series had completely changed its context of usage in this new setting in the AV-Portal of the TIB, where all digitized IWF films are hosted today. Here, the fact that the zeppelin film series appeared in the history section of the IWF is completely omitted.^21^ (Fig. 8)

Thus, with the digitization and online publication of footage material new questions of reuses and reinterpretations arise. One is forced to consider what type of “audiovisual knowledge” is produced by a video platform like the AV-Portal. Is the content arranged in a user-friendly manner? Is the content curated so that a critical viewer might know where to seek out more information? The short answer to these questions is: no. This is in part due to the fact that the platform is not merely made for historical film documents but rather for all sorts of audiovisual content for academic use, of which the IWF Collection is only one part. Malte Hagener has convincingly argued that platforms such as Vimeo, YouTube and Dailymotion disadvantage content that lies beyond the mainstream, including historical video footage, because the algorithms are not optimized to privilege these films (Hagener 2016: 186–187). Their popularity is negligible and according of the logic of those platforms, the less frequently they are searched, the more difficult it is to locate them within the endless number of films. Yet, what we see in the AV-Portal is a shift in context of the film series, notably *G 24* and *G 41*: while Terveen hosted these films within a series of contemporary history footage material, the zeppelin mini-series now appears in the context of the history of technology, which was not at all Terveen’s intention. This altered contextualization is partly due to the fact that the TIB is a technical institution and seeks to address academics with a background in the technical sciences. Nevertheless, presenting historical film documents like the series on airship aviation in Germany demands transparent decisions about both the content and context of the films so that they can be used by film historians, historians of science and academics from other fields alike. For the second time in the history of *Airship Aviation in Germany,* the films are found among films from other disciplines. Initially, within the context of the 1950s film encyclopedia, the zeppelin footage was found alongside biological films on movement and behavior, later it found a home on a platform made for technical films with annotations. However, this presentation is not fully adequate to contextualize the material: an optimal solution would embed the films in a curated platform that provides background information on each of the films, puts the images in dialogue with each other and offers further reading on each piece. Such databases already exist, for example at the Fortunoff Archive^22^, the Visual History of the Holocaust^23^ or the Visual History Archive, located at Stephen Spielberg’s Shoah Foundation.^24^ These film archives provide a useful model for improving the contextualization of Terveen’s *Film Documents* in the future.

## Footnotes

1 I would like to thank Rolf Aurich from the Deutsche Kinemathek Berlin for the very valuable comments that he provided throughout the time when writing this article. I also thank my colleague Paul Feindt from the TIB for his endless patience for my numerous questions in regard of the zeppelin series. Furthermore, I am indebted to Sarine Waltenspül and Mario Schulze (both Zurich University of Applied Arts) who have given me much support throughout the last months and with whom I have discussed my findings many times.

2 https://av.tib.eu/.

3 The films can be found at: 10.3203/IWF/G-24 (signature G 24); 10.3203/IWF/G-19 (signature G 19); 10.3203/IWF/G-25 (signature G 25) and 10.3203/IWF/G-41 (signature G 41). In this text, G 24 and G 41 in particular are discussed in more detail.

4 There are only two works on the history of the RWU that are rather collections of sources than historiographical works (Ewert 1998; Kühn 1998). Especially Ewert 1998 is an often cited but highly problematic work since the author systematically denies all ties between the RWU and the National Socialist Party. However, Schmidt 2002 has proven that this is not true. The publication by Munz (2016) also provides insights into the history of RWU. Also, the overview volume on the history of documentary film in the 3rd Reich, which was delivered by von Keitz 2005 gives insights into the history of the institution, but mainly on the situation of educational films. As far as the processing of the history of the IWF institute is concerned, the literature situation is quite clear. Cf. e.g. (Große 2012). This book contains mainly notes and memoirs of the former IWF staff member, as well as numerous photographs. From a cultural anthropological point of view, (Näser 2013) has already approached the IWF. Sarine Waltenspül and Mario Schulze are studying the history of the IWF, as well as the *Encyclopaedia Cinematographica* in depth in their forthcoming article (Schulze & Waltenspül 2021).

5 A quotation from Wolf, which looks back on the history of the Institute, is very revealing here, precisely because it remains so vague in its statement: “When we set about systematically building up our work 25 years ago, scientific film had a hard time. It was not accepted as a method by the representatives of the exact sciences. […] We drew the consequence. The scientific film had to be further developed into a scientific method. […] The entire work of the Institute for Scientific Film was based on this strict view of film as a research method; precisely because of this, it also became a centre of international cooperation.” (Wolf 1975: 7)

6 The historical relationship between the various institutions IWF and RWU is complex and has not been the subject of historical analysis. A first overview is provided by Aurich (1989). Juliane Scholz is currently working on a publication on this topic at the Max Planck Institute for the History of Science. An explanation of the more precise connections would go too far in this context, it should only be mentioned here that the IWF existed as part of the FWU in the rooms of the former Kaiser Wilhelm Institute for Flow Research before its spin-off in 1956. Cf. Scholz 2019: The archives of the Max Planck Society contain numerous founding documents of the IWF, listed below as “AMPG, Repositur, Nummer.”

7 Neither on the history of the IWF nor on the EC there is a sound scientific study available. First findings have already been compiled by Sophia Gräfe in a short text, cf. (Gräfe 2018). In addition, Vinzenz Hediger has recently dealt with the EC in detail, especially with regard to the work of Irenäus Eibl-Eibesfeld (Hediger 2017). The archives of the EC (as well as of the IWF) are located in the Technische Informationsbibliothek Hannover. While the film holdings are indexed and listed in the TIB’s AV portal (https://av.tib.eu/), the written material has not yet been listed and is available in over 3,000 folders in the depot in Hannover/Rethen.

8 10.3203/IWF/G-24

9 O.N. Merkblatt zur Benutzung der historischen Filmdokumente aus der Encyclopaedia Cinematographica des Instituts für den Wissenschaftlichen Film Göttingen. AMPG, Repositur 102, Nr. 208,2 Bl. 394.

10 Merkblatt, AMPG, Repositur 102, Nr. 208,2 Bl. 394.

11 Merkblatt, AMPG, Repositur 102, Nr. 208,2 Bl. 393.

12 Merkblatt, AMPG, Repositur 102, Nr. 208,2 Bl. 393.

13 Terveen, Friedrich o. D. Was heißt wissenschaftliche Enzyklopädie und was ist eine enzyklopädische Einheit? (Unveröffentlichtes Manuskript). In: DKB, Nachlass Friedrich Terveen. O.N. Terveen’s estate, hereinafter referred to as “DKB, Nachlass Terveen,” is currently available in an unordered or unlisted form as it was handed over to the Deutsche Kinemathek Berlin by Terveen’s widow in 2000. It consists of a total of 11 files with correspondence, manuscripts, seminar drafts and photos.

14 Cf. also Albert Fidelius’ letter to Fritz Terveen, dated 5 October 1955, Deutsche Kinemathek Berlin, Nachlass Friedrich Terveen. O.N.

15 “Protokoll über die Sitzung des erweiterten Beirats es IWFs vom 5.12.1958,” Archiv der Max-Planck-Gesellschaft, Abt. II, Repositur 102, Nr. 209,1, Bl. 41, as well as the statistics compiled by Terveen, which are handed down in his *Nachlass*: Deutsche Kinemathek Berlin, Nachlass Friedrich Terveen. O.N.

16 Letter from Friedrich Terveen to Eberhard Lutze (Minister for Science and Culture of Bremen at the time), dated January 6, 1960. O.N.

17 Letters from Friedrich Terveen to Willi Mohaupt from March 23, 1959 and from February 11, 1960. DKB, Nachlass Friedrich Terveen. O.N.

18 Letter from Friedrich Terveen to Wolfgang Kiepenheuer dated January 30, 1960. DKB, Nachlass Friedrich Terveen. O.N.

19 Terveen, Friedrich 1958. “Niederschrift über einige Bearbeitungs- und Veröffentlichungsgesichtspunkte bei der Publikation von historischen Filmdokumenten aus nationalsozialistischer Zeit”. Unpublished manuscript. In: DKB, Nachlass Terveen, O.N.

20 This is revealed in numerous letters from Terveen to Kiepenheuer in the DKB, Nachlass Friedrich Terveen.

21 The zeppelin series does not appear in the history catalogue from 1984 on. By creating the classification codes for the TIB online-databases, the print classifications were often not applicable and were changed.

22 https://fortunoff.library.yale.edu/ (last accessed 09.2.2021).

23 https://www.vhh-project.eu/ (last accessed 09.2.2021).

24 http://sfi.usc.edu/ (last accessed 09.2.2021).
